# Supplementary figures and images for: PKR modulates sterile systemic inflammation-triggered neuroinflammation and brain glucose metabolism disturbances
Source: Front Immunol. 2025 Feb 25;16:1469737. doi: 10.3389/fimmu.2025.1469737 (PMC11893411; doi:10.3389/fimmu.2025.1469737)

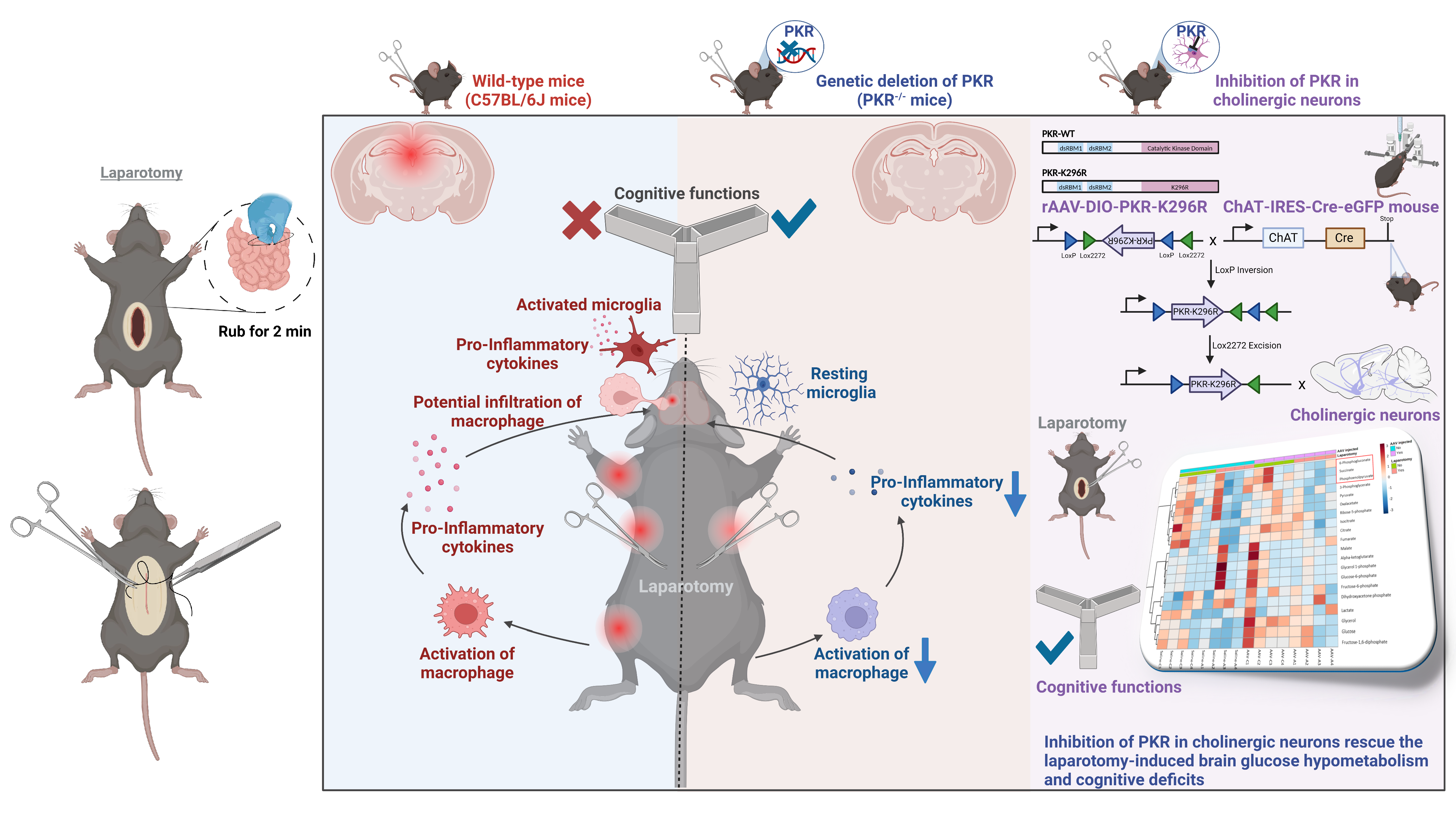

Supplement: Supplementary file 2 [file Image1.tiff]
